# Biodegradable Polymer Membranes Applied in Guided Bone/Tissue Regeneration: A Review

**DOI:** 10.3390/polym8040115

**Published:** 2016-03-29

**Authors:** Jiaolong Wang, Lina Wang, Ziyu Zhou, Hanjian Lai, Pan Xu, Lan Liao, Junchao Wei

**Affiliations:** 1 Department of Prosthodontics, Affiliated Stomatological Hospital of Nanchang University, Nanchang 330006, China; 406531513386@email.ncu.edu.cn (J.W.); 406531514649@email.ncu.edu.cn (Z.Z.); 2 College of Chemistry, Nanchang University, Nanchang 330031, China; 5901213058@email.ncu.edu.cn (H.L.); 5503113042@email.ncu.edu.cn (P.X.); 3 College of Science, Nanchang Institute of Technology, Nanchang 330029, China; linawang@nit.edu.cn

**Keywords:** biodegradable polymer, GTR, GBR, membrane, collagen, polylactide

## Abstract

Polymer membranes have been widely used in guided tissue regeneration (GTR) and guided bone regeneration (GBR). In this review, various commercially available membranes are described. Much attention is paid to the recent development of biodegradable polymers applied in GTR and GBR, and the important issues of biodegradable polymeric membranes, including their classification, latest experimental research and clinical applications, as well as their main challenges are addressed. Herein, natural polymers, synthetic polymers and their blends are all introduced. Pure polymer membranes are biodegradable and biocompatible, but they lack special properties such as antibacterial properties, osteoconductivity, and thus polymer membranes loaded with functional materials such as antibacterial agents and growth factors show many more advantages and have also been introduced in this review. Despite there still being complaints about polymer membranes, such as their low mechanical properties, uncontrollable degradation speed and some other drawbacks, these problems will undoubtedly be conquered and biodegradable polymers will have more applications in GTR and GBR.

## 1. Introduction

Guided tissue regeneration (GTR) was first described in the 1950s by Hurley, who physically separated soft tissues from areas of active bone formation in the spine with a barrier membrane [1]. In the 1980s, GTR was introduced to periodontal tissue regeneration to stop cell migration from gingival connective tissue and epithelium to the periodontal defect, and has been adopted in treating periodontal lesions to generate new attachments [2,3]. Subsequently, a membrane technique used to generate new bone around implants based on the principle of GTR was defined as guided bone regeneration (GBR) [4]. Currently, GBR is one of the most common and promising augmentation techniques to regain sufficient width and height of the jawbone at oral implant sites, or to preserve alveolar sockets after tooth extraction [5,6,7,8]. For GTR and GBR techniques, whether or not the graft material is filled, a special barrier membrane plays a key role to prevent epithelial or undesirable tissues migration into the defective area [9], and consequently it allows sufficient time for bone, cementum, and periodontal ligament regeneration [10]. The ideal membrane for periodontal guided tissue and bone regeneration should have the following properties: biocompatibility, space maintenance ability, cell occlusiveness, integrated by the host tissues, and clinical manageability [11,12]. Generally, the membranes used in GTR and GBR are roughly divided into two types: bioabsorbable and non-resorbable membrane. Each membrane has been extensively applied in clinic. Currently, much attention is still paid to the development of the new types of ideal GBR and GTR membrane [9].

Non-resorbable membranes include expanded polytetrafluoraethylene (e-PTFE, Gore-Tex^®^), high-density polytetrafluoraethylene (d-PTFE), and titanium-reinforced high-density polytetrafluoraethylene (Ti-d-PTFE) membranes [13]. The e-PTFE membranes have been accepted as the gold standard materials with excellent biocompatibility, leading to significant bone regeneration in numerous clinical studies. However, stiff e-PTFE membranes may result in soft tissue dehiscence, and thus make themselves susceptible to exposure with subsequent progression of infection [14]. The following commercially dense PTFE membranes might be impervious to bacteria, since their porosity is less than 0.3 microns. Besides, titanium frame made the Ti-d-PTFE membranes able to be trimmed to desired shapes, and shaped for tenting and space maintenance (Table 1) [13]. However, a second surgery is still necessary to remove them. Bioabsorbable membranes have the advantage of not requiring surgical removal. The main challenge of bioabsorbable membranes is to match its resorption time with the periods of tissue formation. The structural integrity of the membrane should be maintained during the maturation of the newly formed tissue and it varies according to the application, *i.e.*, 4–6 weeks for GTR for bone and periodontal ligament cells to fill the space but ≥6 months for GBR to support new bone formation and maturation [15]. Hence, an optimal persistence and stability of membranes *in vivo* should be guaranteed in the range from four weeks to several months. In this review, the recent progress of bioabsorbable membranes used in GTR and GBR is reviewed. Depending on their origins, they can be sorted into natural polymers, such as xenogeneic-derived collagen, and synthetic polymer materials, for example, poly(lactic acid), and polymer composites, which refer to a combination of two or more different materials to obtain specific mechanical, chemical, and physical properties [16].

## 2. Resorbable Membranes Based on Natural Polymer

Natural polymers exhibit good biocompatibility, safety, biodegradability, and therefore have gained much attention as GTR and GBR materials. More importantly, their inherent bioactivity, the ability to present receptor-binding ligands to cells, susceptibility to cell-triggered proteolytic degradation and natural remodeling, are advantageous properties compared to synthetic polymers [17]. However, the inherent bioactivity of these natural polymers has its own downsides, including a strong immunogenic response associated with most natural polymers, complexities associated with their purification and the possibility of disease transmission [17]. Collagen and chitosan are the most frequently studied natural polymers for GTR and GBR applications, especially collagen membrane.

### 2.1. Membrane Based on Collagen

Collagen membranes, mostly types I and III, have several superior properties such as good tissue integration, fast vascularization, biodegradation without foreign-body reaction, chemotactic action for fibroblasts, hemostatic property, weak immunogenicity, osteoblastic adhesion and their proven biocompatibility and capability of promoting wound healing. Therefore, collagen membranes attract much interest in GTR and GBR research [15,18,19,20,21,22,23].

Different types of commercially collagen membranes, such as Bio-Gide^®^, Ossix^®^, Biomend^®^ and BiomendExtend^®^, varying from collagen types, physical or chemical structures, have been designed (Table 2). These collagen membranes can be resorbed via enzymatic degradation by collagenases/proteases, and macrophage/polymorphonuclear leukocyte-derived enzymes [14], and bacterial proteases [24,25]. For example, Bio-Gide^®^ (GeistlichPharma AG, Wolhusen, Switzerland), one of the most important commercial collagen membrane, is composed of porcine type I and type III collagen fibers. It comprises a bilayer structure with an outer compact smooth layer and an inner porous layer. When used for GBR, the porous and compact layers can not only enable osteogenic cell migration to make bone ingrowth possible, but also prevent the invasion of fibroblasts [26]. It was observed that Bio-Gide^®^ collagen membranes rapidly adsorb the TGF-β activity released from autogenous bone chips, a molecular process that might contribute to guided bone regeneration [27]. Besides, the degradation of monolayer Bio-Gide^®^ and bilayer Bio-Gide^®^ had no difference [14].

**Figure 1:**
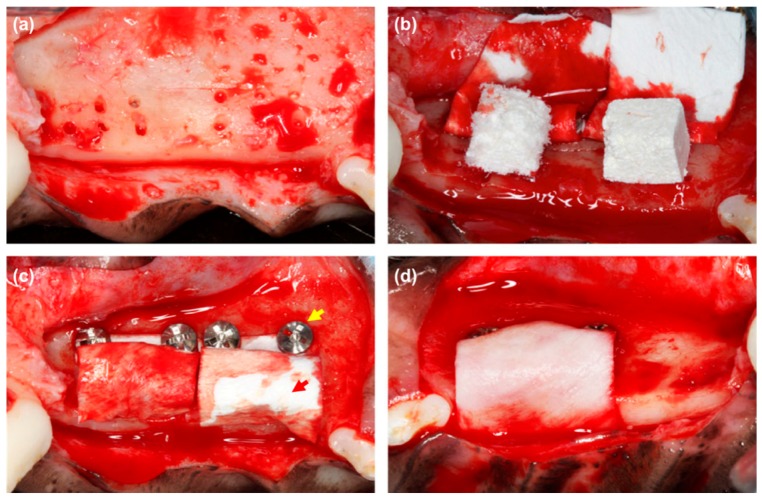
Clinical photographs of the experimental sites: (**a**) A full-thickness flap was elevated, and the bone bed was prepared by perforating the cortical bone; (**b**) Assigned bone substitutes and membranes were applied. The left block is bovine hydroxyapatite incorporated into a non-cross-linked collagen matrix, and the right block is porcine hydroxyapatite incorporated into a cross-linked collagen matrix; (**c**) The bone substitutes were covered by the membranes (red arrow), which were stabilized using two pins (yellow arrow). Both membranes are cross-linked collagen membrane; (**d**) An occlusal view of the opposite side. The non-cross-linked collagen membrane is applied. Reprinted with permission from John Wiley and Sons [40].

Although these native collagen membranes have excellent cell affinity and bio-compatibility [28], and similar bone regeneration capacity to that of non-resorbable membrane [29], they have obvious drawbacks for GTR and GBR applications, including the loss of space-maintaining ability in humid conditions [14,30], risks of a disease transmission to human for animal-derived collagen [31], inferior mechanical strength, and too rapid biodegradation [32]. It has also raised certain ethical and cultural issues [33]. These limitations, such as poor mechanical properties and rapid degradation, are associated with the shortened functional period, greater susceptibility to infection, and the regeneration of new tissue [34,35]. Hence, in order to reinforce the mechanical and biodegradable stability to comprise the biocompatibility for use as GBR and GTR membranes, various chemical, physical, and biological cross-linking methods have been introduced to cross-link collagen. Among the chemical cross-linkers, glutaraldehyde (GTA), 1-ethyl-3-(3-dimethylaminopropyl) carbodiimide (EDC), polyepoxy, diphenyl-phosphorylationazide, *etc.* are the most commonly used. For instance, BioMend^®^ (BioMend Extend^®^) (Zimmer Biomet, Inc., Carlsbad, CA, USA) and Rapi-Gide^®^ (DalimTissen, Seoul, Korea), commercially available membranes, are cross-linked by GTA and EDC, respectively. Via cross-linking, the tensile strength of collagen was enhanced and their degradation time may be prolonged [36]. However, the residual reagents or secondary products during collagen implant degradation may have toxic effects, and thus limit their applications [32]. In addition, certain polysaccharides have also shown a degree of success to cross-link collagen membrane [36,37], such as Ossix Plus^®^ (Datum Dental Ltd., Lod, Israel) membrane. Physical treatments such as dehydrothermal treatment [38,39,40], heat treatment, ultraviolet irradiation, gamma irradiation and microwave irradiation, and biological methods (e.g., transglutaminase) may be used as an alternative to introduce cross-link efficiently [32,41]. Cross-linked collagen membrane can maintain block bone substitutes dimensionally stable in comparison with the use of non-cross-linked collagen in the early healing period of lateral onlay graft (Figure 1) [40].

Although cross-linking of collagen endows it with more advantages, there still are some problems with cross-linked collagen; for example, cross-linked membranes display prolonged membrane integrity with surrounding tissues and blood vessels compared with the non-cross-linked membranes [42]. These chemically and enzymatically cross-linked collagen membranes showed delayed angiogenesis in rats and dogs [29,40]. Some research showed that chemically cross-linked collagen membrane demonstrated more adverse events and insufficient bone regeneration compared to the non-cross-linked collagen membrane [43]. All in all, how to balance the contrary sides of cross-linking collagen membranes between stability and functional remodeling [41] through the considerable complexity and diversity in their structure, their linking degrees, their assembly and their function remains a challenge.

### 2.2. Membrane Based on Chitosan

In the past 20 years, chitosan, 1,4-linked 2-amino-2-deoxy β-d-glucan, an alkaline linear and cationic polysaccharide obtained from the deacetylation of chitin, has been shown to be an attractive candidate material for GTR and GBR membranes due to its low cost, superior biocompatibility, non-antigenicity, appropriate degradation rate, flexibility in hydrated environments, hemostatic activity, antimicrobial and wound healing potential [44,45,46,47]. Chitosan membranes were compatible with cells *in vitro* and able to facilitate bone regeneration in rat calvarial defects [48]. Chemical cross-linking is an effective method to increase its mechanical strength and reduce its degradation speed [49]. Chitosan membranes cross-linked with genipin showed less inflammatory reaction and resulted in faster healing times when compared with GTA [50]. Histological observations show that most non-cross-linked and genipin-cross-linked chitosan membranes became infiltrated by fibrous tissue by 16 and 20 weeks, respectively, whereas BioMend Extend^®^ collagen membranes showed an much earlier infiltration timing at the 12-week time point [49]. It was reported that genipin-cross-linked chitosan electrospun mats exhibited only 22% degradation after 16 weeks *in vitro* test, which was much slower compared to 34% degradation for non-cross-linked mats [51]. Besides, the ultimate tensile strength of the cross-linked mats was 32 MPa, about 165% higher than that of the non-cross-linked mats [51]. These results suggest that genipin-cross-linked chitosan membranes might have potential to meet the clinical requirements for GBR applications.

Another attractive characteristic is its inherent antibacterial property; therefore, chitosan is also widely used as an antibacterial agent, either alone or blended with other natural polymers [52]. However, research on its antibacterial application in GBR and GTR is scarce. Chitosan nanoparticles could adhere to mucosal surfaces, and thus prolong the residence time and evaluate drug permeation at drug absorption sites [53]. It was reported that chitosan nanoparticles acted synergistically with chlorhexidine in collagen membranes for periapical guided tissue regeneration [53]. These results suggested that antibacterial property of chitosan could be used to improve regenerative procedures in periapical surgery.

### 2.3. Membrane Based on Gelatin

Gelatin, a soluble protein derived from partially denatured collagen, has received great attention owing to its availability, easy handling and cost efficiency [54]. Attractive properties of gelatin, such as good biocompatibility, low immunogenicity, plasticity, adhesiveness, promotion of cell adhesion and growth, and low cost, make it ideally suitable as a biomaterial for tissue engineering, GBR and GTR [55]. However, gelatin exhibits poor mechanical properties and fast degradation. An efficient method to improve its mechanical properties and stability is to cross-link gelatin with EDC and *N*-hydroxyl succinimide (NHS) [56], heat treatment [57], and GTA [58]. Although the tensile properties of the gelatin fibrous membrane can be greatly enhanced by cross-linking with EDC/NHS, the cross-linked membranes in the moist state showed a high elastic characteristic but an extremely lower Young’s modulus [56]. Therefore, gelatin is seldom used alone to function as a GBR and GTR membrane.

### 2.4. Membrane Based on Silk Fibroin (SF)

Silk fibroin (SF), a natural protein that can be extracted from silk worms (e.g., *Bombyx mori*) or spiders [59], has many attractive properties, including good biocompatibility, good oxygen and water vapor permeability, and biodegradability [60], and thus has been a candidate material for bone and periodontal regenerative applications. A recent study reported that in the calvarial defect of rabbits with SF nanofiber membrane, a complete bony union across the defects was observed after eight weeks, while at 12 weeks, the defect can be completely healed with new bone [61]. In addition, SF provides remarkable strength and toughness to provide enough stability, which benefits for space maintenance for bone ingrowth while preventing membrane collapse [61]. The tensile strength of the wet SF membrane was higher than the tensile strength of the wet EDC-cross-linked collagen and PTFE membranes. The bone formation of the SF membrane was also higher than those of the other two membranes observed by μ-CT and histological analysis [62]. All these results strongly suggest that the SF membrane could be useful as a barrier membrane for GBR and GTR.

## 3. Resorbable Membranes Based on Synthetic Polymer

Most of current resorbable synthetic polymer membranes on the market are based on aliphatic polyesters, such as poly(lactic acid) (PLA), poly(glycolic acid) (PGA), poly(ε-caprolactone) (PCL), poly(hydroxyl valeric acid), and poly(hydroxyl butyric acid), as well as their copolymers. The use of these membranes may be subject to drawbacks such as inflammatory foreign-body reactions associated with their degradation products [63]. Some studies found a reduced defect fill when applying PLA and PGA membranes as opposed to e-PTFE membranes [64,65]. More importantly, they are generally not as biologically active as natural polymers [17]. However, due to their excellent biocompatibility, controllable biodegradability, low rigidity, manageability, processability, and drug-encapsulating ability [64,66,67,68], they have been widely considered for orthopedic applications both in theoretical experiments and clinic, especially in GBR and GTR procedures.

### 3.1. Polylactic Acid (PLA) and Polylactic acid/Polyglycolic Acid Copolymer (PLGA)

Polylactic acid (PLA) is one of the most common and important polymers used in GTR and GBR procedures because of its suitable mechanical properties and biocompatibility. In order to regulate the degradation rate and hydrophilicity of PLA, copolymers of lactide and ε-caprolactone, glycolide, *etc.* have been synthesized. Polylactic acid/polyglycolic acid copolymer (PLGA) is a well-known alternative for PLA in orthopedic applications. Both PLA and PLGA have been used commercially as membranes, such as Resolut Adapt^®^, Vicryl^®^, Epi-Guide^®^ and Vivosorb^®^, and every membrane may have its own properties (Table 3). For example, Guidor^®^ Matrix Barrier (Sunstar Americas, Inc. near Chicago, IL, USA), the first and most widely studied alloplastic matrix and barrier technology available, is bi-layered, and it is made from a homogenous blend of two polymers, poly-d,l-lactide (PDLLA) and poly-l-lactide (PLLA), doped with acetyl tri-n-butyl citrate [69]. GUIDOR^®^ Matrix Barrier could maintain its barrier function for a minimum of six weeks, while it is gradually resorbed in 13 months [69]. Resolut Adapt^®^ and Resolut Adapt^®^ LT (W.L. Gore and ASSOC, Flagstaff, AZ, USA) membranes were made by PLGA, and they can remain substantially integrity for 8–10 weeks and 16–24 weeks, respectively, in order to meet GBR and GTR different demands.

PLGA membrane have shown similar result in extraction wound healing by GBR protocol compared with collagen membrane [70]. Besides, some study reported that both Gore-Tex^®^ and Resolut Adapt^®^ membranes in combination with bioactive glass were equally effective in enhancing the periodontal regeneration [71]. However, in a randomized controlled trial, PLGA membrane can not maintain the horizontal thickness of regenerated bone as well as Ti-e-PTFE membrane, and the latter membrane revealed less soft tissue complications [72].

PLA and PLGA membrane prepared by most fabrication techniques were stiff, which impeded their medical applications [73], while this drawback can be solved by introduction of softeners, such as *N*-methyl-2-pyrrolidone (NMP). Some studies recently have shown that NMP could soften PLGA membranes and accelerate the maturation of preosteoblastic cells and bone regeneration [73,74,75,76]. When the NMP released, the membrane would turn re-stiffness again. Besides, its contents have a positive role on these PLGA membranes with regard to bone ingrowth [76]. When combined with deproteinized bovine bone mineral, this PLGA can perform similarly to native collagen [73]. In addition, 3 wt % lauric acid can provide a remarkable plasticizing effect on PLGA because of weak intermolecular interactions in PLGA, and the elongation at break (16.1%) is much higher than that of pure PLGA (9.1%) [77].

Although PLA- and PLGA-based membranes are non-cytotoxic and biodegradable, the releases of oligomers and acid byproducts during degradation may trigger inflammation reactions and foreign body response *in vivo* [73,78,79], and thus many studies have been carried out to tune its properties, for example, blending with hydroxyapatite (as shown in Section 4.2.).

### 3.2. Polycaprolactone (PCL)

Due to its biocompatibility, low cost and high mechanical strength, polycaprolactone (PCL) is an attractive biomedical polymer and has been extensively studied in bone tissue engineering [80,81,82]. Only a few studies have studied PCL-based GTR membranes [83,84]. PCL does not produce a local acidic environment during the degradation procedure compared with PLA and PLGA. However, the complete bioresorption *in vivo* of PCL membranes is approximately 2–3 years, which is too long for application in GTR and GBR treatment [13]. Furthermore, its hydrophobicity reduces cell adhesion and proliferation. Therefore, PCL is always blended or copolymerized with other polymers before biomedical application (as shown in Section 4.1.).

### 3.3. Polyethylene Glycol (PEG)

Polyethylene glycol (PEG), as an important biodegradable, cell-occlusive, and biocompatible polymer, has also been a candidate for GBR and GTR membranes [85,86,87]. PEG hydrogel showed a high biocompatibility and tissue integration in rats, and degradation was dependent on PEG composition [86]. In a randomized controlled trial, PEG hydrogel membrane was as successful as collagen membrane in the treatment of peri-implant bony dehiscences with simplified clinical handling [87]. In addition, recent studies found that PEG membranes exhibited perspective potential for staged augmentation of challenging lateral ridge defects and preservation of the ridge contours [23,88,89,90].

## 4. Resorbable Membranes Based on Polymer Composites

### 4.1. Polymer Blends

As polymer membranes have several essential criteria for GBR and GTR success, including biocompatibility, proper degradation profiles, adequate mechanical and physical properties, and sufficient strength to avoid membrane collapse and assure sufficient barrier function [12], single polymer cannot meet all the criteria. For example, natural polymers always lack sufficient mechanical strength and degradation profiles, while synthetic polymers are biologically inert. It has been reported that some polyester-based membranes become stiffer and brittler after placement in PBS or artificial saliva solution [91]. It is still a challenge to develop membranes with sufficient mechanical properties, predictable degradation rate, and structure that mimics closely the native extra cellular matrix (ECM) [92]. It may be an efficient solution to blend two kinds or more of polymers to hinder their respective limitations and show more positively synergistic effects.

#### 4.1.1. Blends of Natural Polymers

Although chitosan is natural polymer, its bioactivity was not as well as protein polymers, and its mechanical properties are poor. Many works have been carried out by blending chitosan with other polymers to improve the mechanical properties and bioactivity of scaffolds composed of several components. For example, gelatin contains free carboxyl groups on its backbone, and it is easy to blend with chitosan to form a network by hydrogen bonding. It was shown that the ability to support cell adhesion and proliferation of gelatin/chitosan membranes was better than gelatin or chitosan alone [93]. Besides, after proanthocyanidin cross-linked, gelatin/chitosan became more stable and possessed higher mechanical properties compared with gelatin and chitosan/gelatin membranes [93]. A tri-layered membrane with a central chitosan layer sandwiched by two collagen membranes containing 20 wt % HA was fabricated [94]. The hydroxyapatite/chitosan/gelatin membranes not only promote human bone marrow mesenchymal stem cells (hBMSCs) proliferation, but also enhance progression of osteogenic differentiation [95]. These results suggest that such gelatin/chitosan or collagen/chitosan membranes are promising candidate for guided tissue and bone regeneration applications which possess sufficient mechanical and structural properties to function as a barrier membrane, and that the proteins promoted osteogenic differentiation.

#### 4.1.2. Blends of Synthetic Polymers

PLGA is well known for having a good influence on the reconstruction of various tissues because of its superior cytocompatibility. However, because of its weak mechanical strength, it is hard to maintain the shape of a PLGA scaffold during various *in vitro* and *in vivo* experiments. Thus, PLGA have been blended with other polymers, for example, PCL/PLGA composite scaffolds were manufactured by mixing PCL and PLGA in the same ratio, and their compressive strength and modulus were much higher than that of pure PLGA scaffolds [96].

A series of PDLLA/PLGA electrospinning membrane system with appropriate degradation rate and excellent cell-occlusiveness were prepared for GTR, and the *in vitro* cytologic research revealed that PDLLA/PLGA composite membranes could efficiently inhibit the infiltration of human embryonic kidney 293T cells. Besides, the subcutaneous implant test on Sprague-Dawley (SD) rat showed that PDLLA/PLGA (70/30, 50/50) composite membranes could function well as a physical barrier to prevent cellular infiltration within 13 weeks, implying that PDLLA/PLGA composite membranes could serve as a promising barrier membrane for guided tissue regeneration [92]. Besides, PLA/PCL, PLGA/PCL and many other synthetic polymer composites may also have bright future in GBR and GTR [96,97,98].

#### 4.1.3. Blends of Natural Polymer and Synthetic Polymer

Natural polymers always possess much better biocompatibility or bioactive properties, for example gelatin, has many integrin-binding sites for cell adhesion and differentiation [99,100]. When blend the natural polymers with synthetic polymers, it may combine both the advantages of natural and synthetic polymers. PCL-gelatin blend, a new biomaterial with good biocompatibility and improved mechanical, physical, and chemical properties, has been successfully used in neural tissue engineering [101], cartilage tissue engineering [102,103], GBR and GTR applications [99,100,104,105,106]. However, phase separation between PCL and gelatin is a headache to prepare composites with excellent resultant performance. Acetic acid could effectively mediate the miscibility of PCL and gelatin, and thus it was generally used to form homogeneous nanofibers with improved performance [106,107]. The biodegradation time of the membranes was also appropriate for tissue regeneration [106].

**Figure 2:**
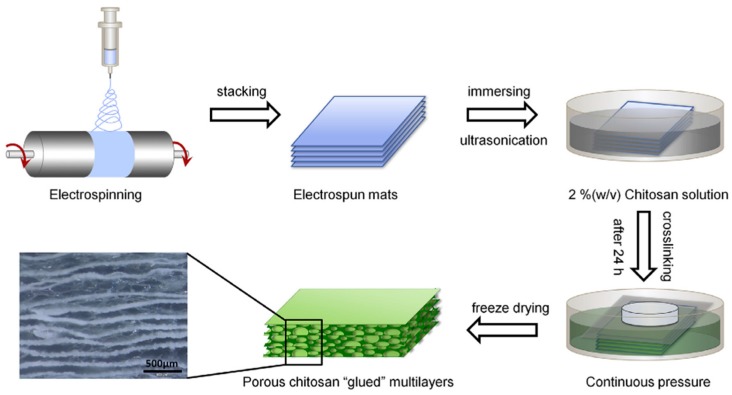
The schematic process of fabricating PCL–PEG nanofibers embedded 3D scaffold by incorporating PCL–PEG nanofibrous mats (aligned or random) into porous chitosan scaffold. The optical image displays a representative section view of the scaffold with width of 103.38 ± 49.54 µm between layers. Reprinted with permission from Elsevier [109].

Many chitosan-based hybrid systems have been prepared to increase the cell adhesion, proliferation, and differentiation ability of polyester membranes or scaffolds. When compared to pure PLLA electrospun membrane, PLLA/chitosan electrospun composite membranes showed more potential for clinical application due to their faster degradation rate and non-fibroblast penetration property. The degradation rate was up to 20% in six weeks, while PLLA electrospun membrane was almost non-degradable [108]. Aligned PCL–PEG nanofibers were incorporated into porous chitosan scaffolds to improve the orientation of collagen fibers in regenerated periodontium (Figure 2) [109]. Ku *et al.* [110] designed PLLA/chitosan multilayered membrane composed of the outer layers of chitosan mesh for ease of cell adherence and the middle layer of nanoporous PLLA for sufficient mechanical strength. The membrane maintained its integrity for up to eight weeks while allowing gradual degradation. These results suggest that these chitosan/polyester membranes may be suitable for use in GBR and GTR.

### 4.2. Bio-Ceramic/Polymer Composites

In order to develop attractive biomaterials for GBR and GTR, considerable attention has been paid to biomimetic bone extracellular matrix (ECM) structure, composites of polymer and bio-ceramics component [111]. The latter component refers to hydroxyapatite (HA) [112], carbonated hydroxyapatite (CHA) [113], bioactive glass (BG) [114], β-calcium phosphate (β-TCP) [115,116] and so on, which is known for its good osteoinductive, osteoconductive properties and excellent biocompatibility [117]. Among the available bioactive ceramics, except for its superior osteogenic and angiogenic effects [118,119], BG can regenerate not only hard tissues, but also soft tissues [120], and thus BG has received increasing attention in periodontal regeneration, since the periodontal tissues consist of both hard tissues (*i.e.*, cementum and alveolar bone) and soft tissues (*i.e.*, gingiva and PDL). It has been shown that the incorporation of bioactive ceramics can significantly enhance mineralization and cell activities on polymer membranes, indicating favorable osteoconductivity and/or osteoinductivity for GTR and GBR applications [45,83,114,121,122]. Besides, these bioactive materials can improve the mechanical properties [123]. The 10–30 wt % nanoapatite in the membrane demonstrated higher tensile strength (0.61 MPa) compared with pure PLGA (0.49 MPa) [77]. Moreover, the addition of bio-ceramics can neutralize the acidic degradation products from the polymers such as PLA and chitosan by the alkali groups [45,47,124]. These composite membranes are assumed to have the ability to preserve the structural and biological functions of the damaged hard tissues in a more efficient and biomimetic way [125]. Zinc HA powders were introduced into a heat treated cross-linked gelatin membrane, which exhibited greater bone formation than collagen membrane did. It was Zinc HA that stimulated bone formation through the actions of zinc ions accelerating the proliferation and differentiation of osteogenic cell [57,126]. A biomimetic coating of apatite on a collagen template can be considered an efficient alternative [127]. Biomimetic precipitation process has been used to form apatite coating on collagen formulations [128,129,130]. This emphasizes the need for polymer/bio-ceramics composite materials that can combine the advantage of both materials.

## 5. Resorbable Membranes Containing Functional Materials

### 5.1. Polymer Membranes Loaded with Antibacterial Agents

The development of periodontitis is mainly related to bacteria activities. Moreover, bacterial infections are currently considered to be the major reason for the failure of GTR and GBR membranes in clinical applications. Antibacterial properties attached to GTR and GBR membranes is one of the greatest interests in the war against implant-related infections, representing the broadest group of anti-infective biomaterials [131]. Membranes loaded with antibiotics have been designed for local drug release to overcome the adverse effects of conventional systemic drug administration. For example, metronidazole (MNA)-loaded polymeric membrane showed a significant improvement on the periodontal and bone regeneration following GTR and GBT [100,106,107,132]. The PCL/gelatin electrospun membrane loaded with 30% MNA had the best comprehensive properties including superior biocompatibility and antibacterial ability [100]. Moreover, acetic acid was introduced to effectively connect strong interaction between PCL, gelatin, and MNA [106,107]. The controlled and sustained release of MNA from the membranes significantly prevented the colonization of anaerobic bacteria [106].

In additional to antibiotics, non-antibiotic antibacterial agents also possess a superior antibacterial ability and have been used in GTR and GBR. Lauric acid loaded PLGA-CaP hybrid membranes [77] and ZnO-loaded PCL or PCL/gelatin electrospun membranes [133] have been designed for GTR and GBR. Chitosan nanoparticles and chlorhexidine have also been added in collagen membranes to endow antibacterial activity for periapical guided tissue regeneration [53].

Generally, antibiotics were directly blended with membranes, resulting in a high burst release and short release period that could not effectively prevent bacterial infections. Hence, it is important to develop novel GTR and GBR membranes with sustained and controlled release of antibacterial agents, especially when used for patients with a predisposition to these kinds of complications: smokers, patients with diabetes mellitus, and so on [134,135,136].

### 5.2. Polymer Membranes Loaded with Growth Factors

**Figure 3:**
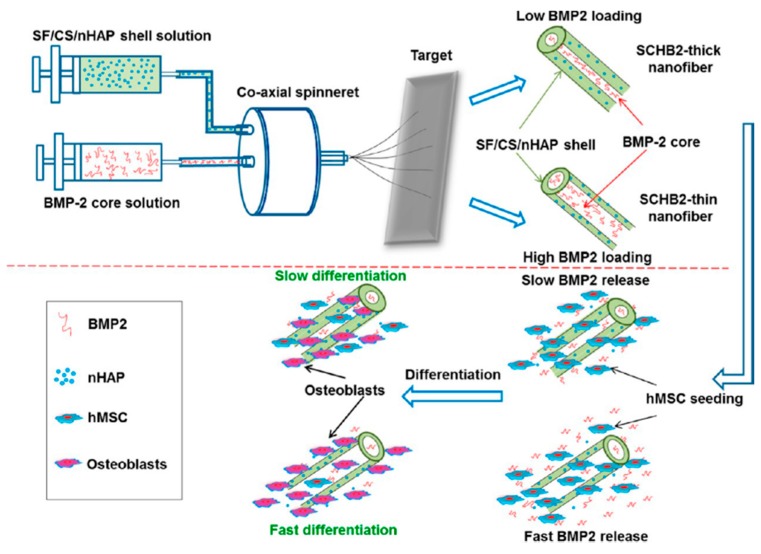
Schematic Representation of the Preparation of SCHB2-Thick and SCHB2-Thin Nanofibrous Membranes through Coaxial Electrospinning and Their Influence on human marrow mesenchymal stem cells. Reprinted with permission from American Chemical Society 2015 [140].

Growth factors are critical signaling molecules that instruct cells behavior through binding to specific transmembrane receptors on the target cells, and one may achieve tissue regeneration by enabling control over growth factor delivery [137]. GBR and GTR membrane can act as a localized controlled release system for growth factors, hence to encourage the differentiation of osteogenic progenitor cell types in the secluded space under the membrane [104]. In the past decades, controlled drug delivery systems and biomaterial scaffolds with various osteogenic factors, especially bone morphogenetic proteins (BMPs), are widely used clinically to promote bone regeneration [138]. BMPs have an unparalleled, dose-dependent potential to augment alveolar bone by triggering the angiogenesis, migration, and proliferation of mesenchymal stem cells, and their differentiation to osteoblasts and chondroblasts. Still, the appropriate methods and optimal doses to allow safe use of recombinant human bone morphogenetic protein-2 (rhBMP-2) is a challenge [139]. PCL/PLGA/β-TCP GBR membrane loaded with rhBMP-2 was successfully prepared via 3-D printing method, realizing sustained release of rhBMP-2 up to 28 days; meanwhile, after four and eight weeks *in vivo* test, the implantation of rhBMP-2 loaded membrane induced much more new bone formation and led to almost entire healing of 8 mm calvarial defects within eight weeks [115]. When incorporating BMP-2 in the core and silk fibroin/chitosan/HA as the shell layer of a nanofiber with two different shell thicknesses (SCHB2-thick and SCHB-thin), the release rate of BMP-2 and the concentration of BMP-2 in the release medium were higher for SCHB2-thin membranes because of reduced shell thickness. Compared with SF/CS and SF/CS/HA membranes, BMP-2 obviously promoted osteogenic differentiation of hBMSCs (Figure 3) [140]. Stromal cell-derived factor-1α (SDF-1α) regulates the migration of hBMSCs in a dose-dependent manner [141]. Compared to the bare membranes, SDF-1α loaded membranes yielded a six-fold growth in the amount of bone formation [104]. These membranes with adequate mechanical properties and the capacity to release growth factors with tailor-made kinetics have potential for optimizing clinical application of GBR and GTR.

## 6. Resorbable Membranes Based on Other Polymer

Although a minor application, platelet-rich fibrin (PRF) in a compressed membrane-like form has also been used as a substitute for commercially available barrier membranes in GTR treatment [142]. PRF is composed of a biopolymer fibrin, and acts as a potent source of growth factors to facilitate the tissue regeneration. However, its rapid degradability within two weeks or less at implantation sites, attaches the disadvantages to application in GBT and GTR for sufficient periods of time [143]. Cross-linking treatments, regardless of methodology, can provide resistance against enzyme-dependent degradation while simultaneously sacrificing the bioactivity of the PRF. Thus, appropriate cross-linking treatment may be a solution to make PRF suitable for GBR and GTR, as long as balance its degradability time and sufficient bioactivity. Freshly prepared human PRF was first compressed with dry gauze and subsequently with a hot iron. Kawase *et al.* [143] employed the heat treatment to prepare PRF membrane, which appeared plasmin-resistant and remained stable for longer than 10 days *in vitro* compared to gauze-compressed PRF. This technique reduces the rate of biodegradation without sacrificing its biocompatibility. Therefore, PRF membranes have promising potential applied as a barrier membrane in the GTR treatment.

Salicylic acid-based poly(anhydride-esters) (SAPAE) has been a promising candidate under issue to improve diabetes’ bone or periodontal tissues regeneration [144]. SAPAE have been synthesized by chemically incorporating salicylic acid, a nonsteroidal anti-inflammatory drug, that reduces the production of pro-inflammatory cytokines within a polyanhydride [145]. Subramanian *et al.* [146] tested SAPAE membrane as a physical barrier and localized salicylic acid delivery system to restrict BMP-2 activity to a specified region. The data indicated that SAPAE polymer membranes have potential application in GBR and GTR procedures and as a barrier to excessive bone formation for diabetes.

## 7. Conclusions and Perspectives

Different types of biodegradable membranes are commonly used in GTR and GBR, as barrier devices to isolate the epithelium from bone tissue to favor bone regeneration. In this review, the advantages and disadvantages of polymer membranes were introduced. The non-degradable e-PTFE membranes have disadvantages such as non-resorbability and the need for a second surgical operation, and thus biodegradable membranes including natural and synthetic polymers showed many exciting advantages. Natural polymer membranes have excellent biological properties, such as cell adhesiveness and biodegradability, however, they are characterized by low mechanical strength and short degradation cycle. As a control, membranes based on biodegradable synthetic polymers possess tuned biodegradation, sufficient mechanical strengths, low rigidity, manageability and processability, while their biological activity is generally not as good as natural polymers. They may also be subject to drawbacks including inflammatory foreign-body reactions associated with their degradation products.

Despite the various drawbacks of biodegradable polymers, they are irreplaceable in GTR and GBR. More and more studies will be carried out on biodegradable polymers and their blends or composites. For example, to some extent, polymer membranes often sacrifice their early angiogenesis and osteogenesis. Thus, future works should address how to effectively promote the osteogenic activities of these membranes when long barrier function time is needed, and hence reach the balance among physiochemical, mechanical and biological properties. Bioactive ceramics that can significantly enhance mineralization and cell activities, combined with biodegradable polymers will be researched more and more. In addition, special membranes with various functional materials, such as antibacterial agents and growth factors, and membranes with tunable degradation speed, mechanical properties and controlled release behaviors will be designed accurately according to clinical demand.

## Figures and Tables

**Table 1:** The most commonly used commercially available non-resorbable polymeric membranes.

Commercial Membrane	Materials	Properties	Comments
Gore-Tex	Expanded PTFE	Good space maintainer; Relatively stiff; Handling	Longest clinical experience
High-density Gore-Tex	High-density PTFE	Porosity of less than 0.3 microns creates impervious barrier to bacteria	Most cost-effective; A non-surgical removal when in an open technique
Gore-Tex-Ti	Titanium-reinforced PTFE	Titanium frame may be trimmed and shaped to create additional space for bone growth	Ideal for ridge augmentation and grafting bony defects missing one or more walls

**Table 2:** The most commonly used commercially available resorbable collagen membranes.

Commercial Name (Manufacturer)	Collagen Type	Collagen Source	Resorption Rate
Non-cross-linked collagen membrane			
CollaTape/CollaPlug/CollaCote (Integra LifeSciences Corp., Plainsboro, NJ, USA)	Type I	Bovine tendon	10–14 days
Periogen (Collagen Corporation, Palo Alto, CA, USA)	Type I and III	Bovine dermis	4–8 weeks
Bio-Gide (Geistlich, Wolhusen, Switzerland)	Type I and III	Porcine skin	2–4 weeks
Tutodent (Tutogen Medical GmbH, Neunkirchen, Germany)	Type I	Bovine pericardium	8–16 weeks
Cross-linked collagen membrane			
OsseoGuard (Zimmer Biomet, Inc., Carlsbad, CA, USA)	Type I	Bovine tendon	6–9 months
OsseoGuard Flex (Zimmer Biomet, Inc., Carlsbad, CA, USA)	Type I and III	Bovine dermis	6–9 months
Ossix Plus(Datum Dental Ltd., Lod, Israel)	Type I	Porcine tendon	4–6 months
BioMend (Zimmer Biomet, Inc., Carlsbad, CA, USA)	Type I	Bovine tendon	8 weeks
BioMendExtend (Zimmer Biomet, Inc., Carlsbad, CA, USA)	Type I	Bovine tendon	18 weeks
RCM6 (ACE Surgical Supply Co. Inc., Brockton, MA, USA)	Type I	Bovine tendon	26–38 weeks
Mem-Lok (BioHorizons IPH, Inc., Birmingham, England)	Type I	Bovine tendon	26–38 weeks
Neomem (Citagenix Inc., Montreal, QC, Canada)	Type I	Bovine tendon	26–38 weeks
OssGuide (Bioland, Cheongju, Korea)	Type I	Porcine pericardium	6 months

**Table 3:** The most commonly used commercially available resorbable synthetic polymeric membranes.

Commercial Name (Manufacturer)	Materials	Properties	Function Time	Resorption Rate
Guidor (Sunstar Americas, Inc. near Chicago, IL, USA)	Poly-d,l-lactide and Poly-l-lactide, blended with Acetyl tri-n-butyl Citrate	2-layer	≥6 weeks	13 months
Resolut Adapt (W.L. Gore and ASSOC, Flagstaff, AZ, USA)	Poly-d,l-lactide/Co-glycolide	Good space maintainer	8–10 weeks	5–6 months
Resolut Adapt LT (W.L. Gore and ASSOC, Flagstaff, AZ, USA)	Poly-d,l-lactide/Co-glycolide	Good space maintainer	16–24 weeks	5–6 months
Epi-Guide (Curasan, Inc., Kleinostheim, Germany)	Poly-d,l-lactic acid	3-layer Self-supporting	20 weeks	6–12 months
Vivosorb (Polyganics, Groningen, The Netherlands)	Poly(d,l-lactide-ε-caprolactone)	can also be used as a nerve guide	10 weeks	24 months
